# Enhanced ultrasonic degradation of methylene blue using a catalyst-free dual-frequency treatment

**DOI:** 10.1016/j.ultsonch.2024.106792

**Published:** 2024-02-03

**Authors:** Lukman A. Yusuf, Zeliha Ertekin, Shaun Fletcher, Mark D. Symes

**Affiliations:** a WestCHEM, School of Chemistry, University of Glasgow, University Avenue, Glasgow G12 8QQ, United Kingdom; b Hacettepe University, Faculty of Science, Department of Chemistry, Beytepe 06800 Ankara, Turkey

**Keywords:** Methylene blue, Ultrasonic degradation, Sonication, Cavitation, Acoustic devices

## Abstract

Methylene blue is one of the most common pollutants found in wastewater, primarily due to its widespread use in the dye industry. Consequently, it is imperative to explore environmentally friendly and efficient methods for degrading this pollutant into non-toxic byproducts. While ultrasonic degradation methods in combination with additives or catalysts have proven effective, such additives or catalysts may inadvertently contribute to secondary pollution. Moreover, the preparation of these catalysts imposes an additional burden in terms of effort and cost. To address these issues, this paper introduces a catalyst-free dual-frequency ultrasound degradation approach for methylene blue. The sonochemical quality of the cavitation bubbles is improved using this technique because the bulk solution is populated with two types of bubbles, whose mean sizes are determined by the dual ultrasound frequencies. The findings demonstrate that, under identical acoustic power density conditions, dual-frequency ultrasound consistently outperforms single-frequency modes across all investigated parameters. Furthermore, the larger the difference between the dual frequencies used, the more effective the degradation of methylene blue. Finally, after just 20 min of sonication, a degradation efficiency of 91% was achieved with dual frequencies of 20 and 80 kHz at an acoustic power density of 209.63 ± 6.94 W/L. Consequently, this technique offers an environmentally friendly, catalyst-free, and highly effective method for degrading methylene blue.

## Highlights

• Methylene blue (MB) is one of the most common pollutants found in wastewater.
• A catalyst-free dual-frequency ultrasound degradation approach for MB is reported.
• Dual-frequency ultrasound consistently outperforms single-frequency modes.
• Larger difference between the frequencies gives more effective degradation of MB.

## 1 Introduction

Industrial wastewater from the textile and printing industries contains a range of persistent organic compounds with degrees of toxicity, carcinogenicity, and mutagenicity, including azo dyes, which pose potential hazards to human health and the ecosystem [1]. Azo dyes, which account for approximately 70 % of global dye production annually, contain an azo group (—N=N—) as the chromophore and other functional groups, such as sulfonic and hydroxyl groups [2], [3]. The inherent stability of these compounds in the presence of light and their resistance to microbial degradation renders their direct discharge (or that of their toxic derivatives) into water courses potentially hazardous. The loss of around 20 % of the yearly output of dyes during the dyeing process, equivalent to more than 7 × 10^5^ metric tons, further contributes to the problem [1]. Methylene blue (MB) stands out as a prominent material within the dye industry due to its extensive utilization in the colouring of silk, wool, cotton, and paper, making it one of the most heavily consumed substances in this sector [4], [5]. Hence, it is crucial to provide an environmentally friendly and efficient method for the degradation of methylene blue into non-toxic byproducts before disposal or consumption. Various techniques have been reported in the literature, including adsorption [5], [6], [7], photo-degradation with catalysts [8], oxidative degradation using nanoparticles [9], ultrasound degradation [10], and electrochemical degradation [11]. In this paper, we focus on the ultrasound degradation method for methylene blue due to its environmental friendliness and high efficacy when fully optimized. It is worth noting that much of the existing literature on ultrasound degradation of methylene blue in wastewater involves the use of additional additives in the form of oxidants or catalysts to enhance the effectiveness of the process. For example, Liu et al. [2] discusses the ozonation-assisted degradation of methylene blue in conjunction with ultrasound enhanced by microchannels. Similarly, Li et al. [12] reported on the sonochemical degradation of methylene blue using tetrachloromethane (CCl_4_) and *tert*-butyl alcohol as additives. Furthermore, Shimizu et al. [13] reported the sonocatalytic degradation of methylene blue with TiO_2_ pellets, while Yuan et al. [14] explored the hybrid sonophotocatalytic degradation of methylene blue, employing a highly ordered TiO_2_ nanotube array as a recyclable catalyst. Meanwhile, Karuppusamy et al. [15], uses a nanoflake catalyst of CaMgO_2_ in combination with ultrasound and photocatalysis to degrade methylene blue. Several other studies have employed various ultrasound-assisted nanoparticle photocatalysts for the degradation of methylene blue [16], [17], [18], [19].

While the efficiency of the process has indeed been improved with the use of various catalysts and additives to enhance the sonochemical degradation of methylene blue, it is essential to recognize that these additional substances may potentially function as secondary pollutants if they are not fully recovered or removed. The adverse effects stemming from the prolonged accumulation of these additives cannot be entirely disregarded. Moreover, the added cost and effort required to procure/fabricate most of these catalysts represent additional burdens on the aforementioned processes. One approach to achieving more effective chemical/catalyst-free sonochemical degradation of methylene blue is to enhance the quality of the cavitation bubbles, which serve as the mechanism for methylene blue degradation. This study aims to improve the quality of cavitation bubbles generated by ultrasound sources operating simultaneously at two different frequencies (20 and 37 kHz, and 20 and 80 kHz). To the best of the authors’ knowledge, this marks the first instance of such an approach being employed for the degradation of methylene blue.

Frequency is inversely related to the size of a cavitating bubble as shown in equation (1), and determines the nature (stable/inertia) of its cavitation regime [20]. Generally speaking, the lower the frequency, the more extensive the pyrolysis that takes place in the vapor phase, while higher frequencies promote the production of radicals. When the frequency is increased, the lifetime of a bubble decreases, leading to rapid collapse and an increased chance of radicals being ejected before they have a chance to recombine inside the bubble [21]. It was reported by Petrier et al. [22] that the oxidation rate was observed to be greater at higher frequencies. This observation led to the suggestion that hydroxyl ions escaped from the cavitation bubbles into the bulk of the liquid due to the bubbles’ short lifetimes, thereby facilitating a subsequent chain reaction:

(1) $$f\approx \frac{3}{R}$$

where f is the ultrasound frequency (in Hz) and R is the bubble radius (in m). It should be however noted that in a multi-bubbles scenario, the bubble sizes (radii) are not usually a single value but cover a range of values around the mean value of R.

## 2 Experimental section

### 2.1 Reagents and chemical analysis

**Fig. 1:**
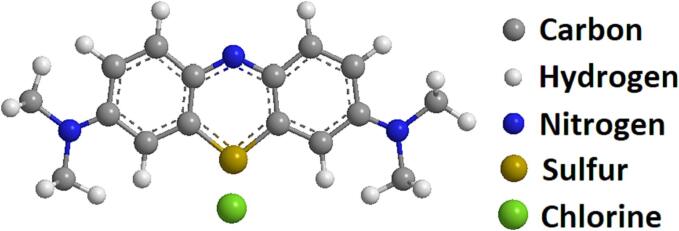
Molecular structure of methylene blue.

The exemplar pollutant utilized in this paper is methylene blue. Methylene blue (CAS No. 122965-43-9) of high purity was purchased from ThermoFisher Scientific. It is an aromatic heterocyclic basic dye with a molecular weight of 319.85 g mol^−1^. It is a well-known cationic and primary thiazine dye with molecular formula C_16_H_18_N_3_ClS and a λ_max_ of 664 nm. It has solubility of 43.6 g/L in aqueous solution at room temperature (25 °C) [23]. Methylene blue belongs to the polymethine dye class and contains an amino autochrome unit, making it a positively charged compound, see Fig. 1. for its molecular structure. According to the International Union of Pure and Applied Chemistry (IUPAC), its chemical name is [7-(dimethylamino)-N,N-dimethyl-3H-phenothiazin-3-iminium chloride], and it is indexed as colour index (CI) 52015 [8].

Five sets of standard methylene blue solution of varying concentration (8 – 41 µM) equivalent to (2.56 – 13.1 mg/L) were prepared and used to obtain a calibration curve, as shown in Fig. S1 in the Supporting Information. Each of the samples was scanned spectrometrically using an Agilent Cary 60 UV–vis spectrometer, and the absorbance values at 664 nm were recorded in each case. A 15 µM solution of methylene blue was then prepared and used as the solution for degradation experiments in the following .

The machine employed for measuring total organic carbon (TOC) was a Thermalox elemental analyzer utilizing thermal oxidation techniques. This process oxidizes the organic carbon within the sample into CO_2._ Subsequently, the CO_2_ is detected and measured using non-dispersive infrared (NDIR), from which the TOC value is derived.

### 2.2 Acoustic set-up

Two types of acoustic device were used in this paper: an ultrasonic horn and an ultrasonic bath. The ultrasonic horn was a commercial Branson 450 W Digital sonifier, operating at 20 kHz through a 230 mm long tapered Ti probe, with a ¼ inch diameter (6.4 mm-ϕ) tip. The ultrasonic bath 320 W (Elmasonic P, Turbex) can be independently operated at 37 kHz and 80 kHz. The input power can be entered manually as a percentage value via the front panel of the control console for each of the acoustic devices.

**Fig. 2:**
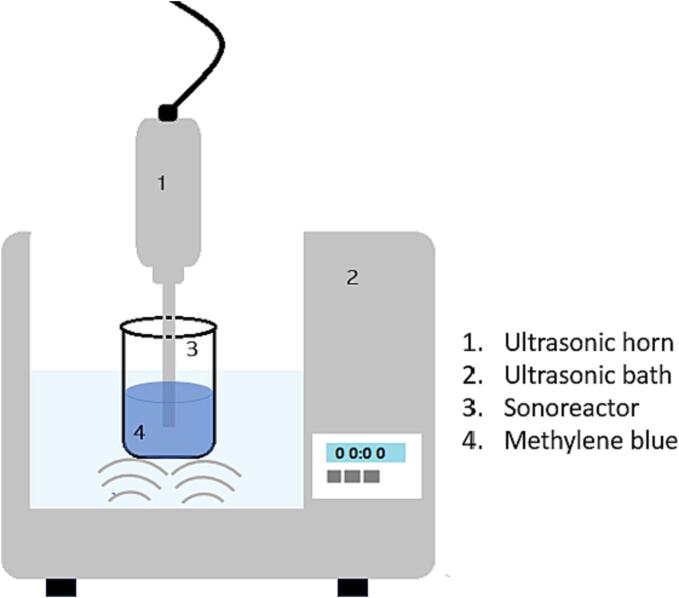
Experimental set up for the degradation of methylene blue. The ultrasonic horn (Branson 450 W) was operating at 20 kHz and the ultrasonic bath (Elmasonic P, Turbex 320 W) was operating at two frequencies (37 and 80 kHz).

The horn was vertically mounted with its tip submerged by 20 ± 1 mm into a 25 mL solution of methylene blue, contained within a custom-made glass sonoreactor (50 mm-ϕ), see Fig. S2 of the Supporting Information. Simultaneously, the sonoreactor was also vertically submerged, positioned roughly 10 ± 1 mm from the base of the ultrasonic bath, which was filled with 600 mL of deionized water, as illustrated in Fig. 2. The position of the horn and other devices remained fixed throughout all the experiments. In all the investigations reported in this work, the temperature of the samples was not maintained at a constant value during ultrasound treatment. We observed a temperature increase in the range of 15–20 °C over the 20-minute sonication period for the dual frequency ultrasound used in this study.

## 3 Results and discussion

### 3.1 Acoustic power calibration

**Fig. 3:**
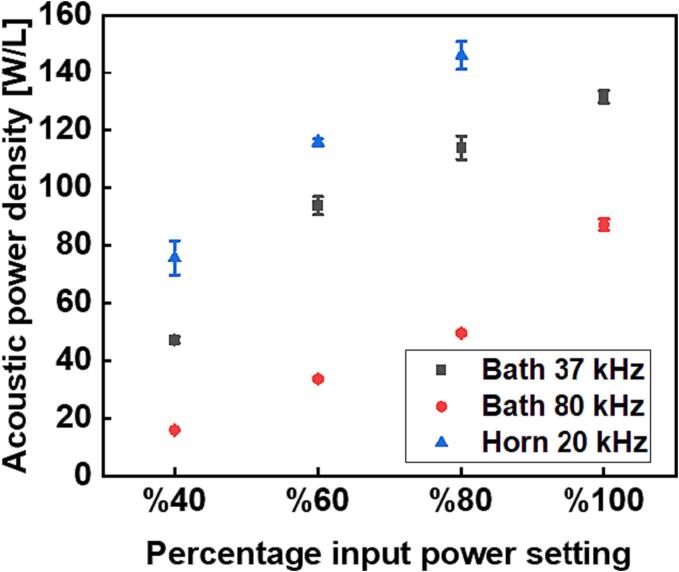
Individual acoustic power density calibration of the acoustic devices operating in continuous mode at some selected percentage input power settings.

At the outset, it is necessary to estimate the acoustic power density generated from each of the acoustic devices at distinct percentage power settings while operating alone or in combination with each other. The acoustic power was estimated using a calorimetric method, where the acoustic power dissipated is proportional to the rate of temperature rise [24]. A thermocouple with a data logger (USB TC-08, Pico Technology) was submerged vertically by a fixed distance of 15 ± 1 mm from the liquid surface into 25 mL of deionized water contained in the sonoreactor and was sonicated continuously for at least 200 s. The thermocouple was held at this position throughout the calibration procedures with the help of a clamp stand. Three sets of readings were taken for each operating setting from which the mean ± standard deviation was computed and plotted. Fig. S3 in the Supplementary Information shows three examples of such calorimetric data. The acoustic power (W) was estimated from the slope of the plot of temperature change against time using equation (2). The acoustic power density was then obtained by dividing the acoustic power by the volume used (25 mL).

(2) $$P_{\mathrm{ac}}=\frac{\mathrm{dT}}{\mathrm{dt}}C_{p}M$$

where $C_{p}$ is the specific heat capacity of water (4.2 J g^−1^ K^−1^), *M* is the mass of water in (g), and dT/dt is the rise in temperature per second. Fig. 3 shows the acoustic power density obtained from each of the acoustic devices while operating alone at some selected power settings. The figure shows that the acoustic power density increased with decreasing operating frequency. The highest power density was obtained from the ultrasonic horn, whose operating frequency was 20 kHz, and the lowest power density was obtained with the ultrasonic bath operating at its highest frequency of 80 kHz. This is because the mean bubble size increases with decreasing frequency and the bubbles collapse more vigorously, hence the acoustic power generated is greater at lower frequency [25], [26].

**Fig. 4:**
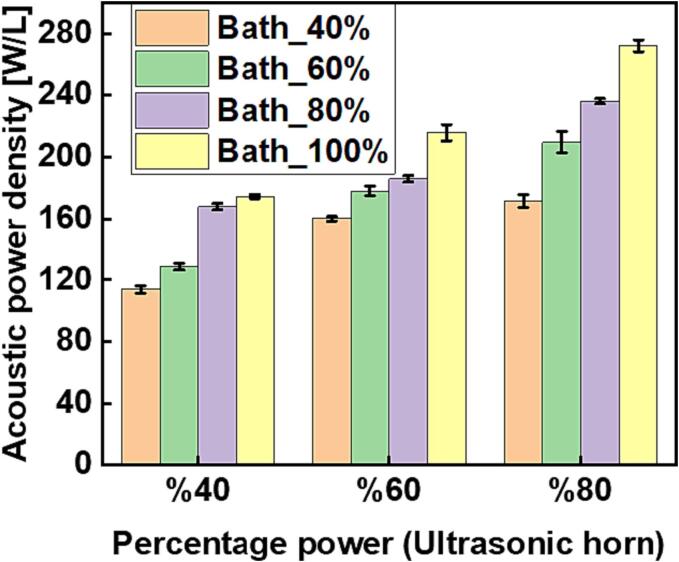
The resultant acoustic power density obtained from simultaneous operation of the ultrasonic horn (20 kHz) and ultrasonic bath (37 kHz) over selected percentage input power settings.

**Fig. 5:**
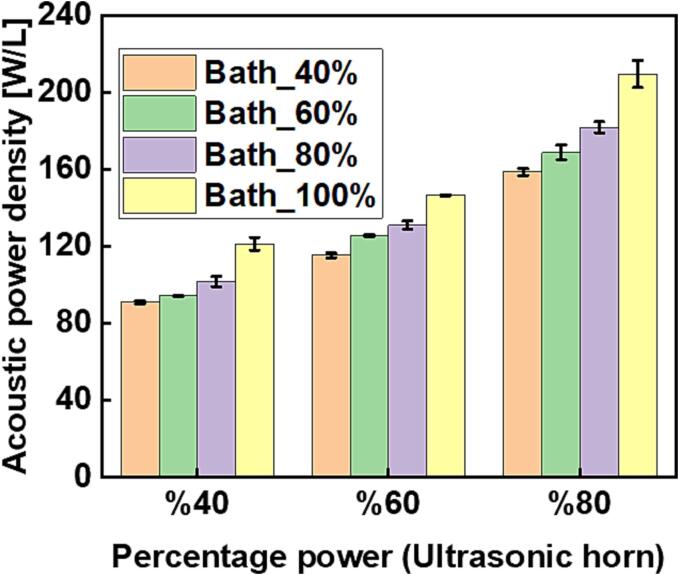
The resultant acoustic power density obtained from simultaneous operation of the ultrasonic horn (20 kHz) and ultrasonic bath (80 kHz) over selected percentage input power settings.

Fig. 4 and Fig. 5 are the respective plots of estimated acoustic power density obtained from operating the two acoustic devices simultaneously at either 20 and 37 kHz (ultrasonic horn and bath respectively) or 20 and 80 kHz (ultrasonic horn and bath respectively) over different combinations of percentage input power settings.

### 3.2 Performance comparison of single and dual ultrasound frequency treatment

Using the acoustic power density data obtained in Section 3.1, three distinct regimes have been identified, as illustrated in Table 1. Each regime encompasses both single and dual operations of acoustic devices at various percentage power settings, yielding approximately equivalent acoustic power densities. The objective is to gain insight into the influence of concurrently activating two acoustic devices operating at different frequencies on the degradation efficiency of methylene blue, as well as to assess the impact of the frequency difference on degradation efficiency. In each case, the methylene blue solution was sonicated for 15 min and the percentage degradation after 15 min was obtained using equation (3).

(3) $$Percentage\;\;degradation=\frac{C_{0}-C}{C_{0}}\times 100\%$$

where *C_0_* and *C* are the initial (before treatment) and final concentrations of methylene blue (after ultrasonic treatment), respectively.

**Table 1:** Comparison of the performance of single and dual frequency ultrasound treatment of methylene blue solutions for 15 min.

**Regime**	**Ultrasonic device/Input power**	**Acoustic power density [W/L]**	**Methylene blue concentration after 15 mins [µM]**	**Percentage degradation rate [%]**
1	Horn_20 kHz/80 %	146.08 ± 4.83	5.64 ± 0.87	62
	Horn_20 kHz/60 % and Bath_80 kHz/ 100 %	146.51 ± 0.26	3.28 ± 0.56	78
2	Horn_20 kHz/60 %	115.48 ± 1.27	6.58 ± 0.64	56
	Horn_20 kHz/40 % and Bath_37 kHz/ 40 %	113.76 ± 2.40	5.94 ± 0.55	60
3	Bath_37 kHz/100 %	131.57 ± 2.10	8.36 ± 0.57	44
	Horn_20 kHz/60 % and Bath_80 kHz/ 80 %	131.13 ± 2.16	4.82 ± 0.56	68
	Horn_20 kHz/40 % and Bath_37 kHz/ 60 %	128.54 ± 2.28	6.29 ± 0.55	58

The results across all regimes reveal two key findings. Firstly, the degradation efficiency of methylene blue exhibited an improvement during dual frequency operation when compared to single frequency operation, as evident in Table 1 for regimes 1–3. This finding shows that the quality of active cavitation bubbles has improved under dual frequency operation because the cavitation zone/sample medium comprises different mean bubble sizes capable of undergoing different cavitation events. The primary cavitation bubbles generated by the ultrasonic horn at 20 kHz typically form a conical structure emanating from the tip, together with few clusters of distal bubbles [27]. The bubbles collapse periodically and generate intense shockwaves leading to higher localized temperatures and pressures at the cavitation site [25]. The cavitation enables the formation of hydrogen peroxide (H_2_O_2_) and promotes chain reactions between the hydroxyl radicals (^●^OH) and the methylene blue molecule. Bubble coalescence (i.e., joining of two or more bubbles) is one of the key phenomena occurring during the cavitation process. During dual frequency sonication, the combination of high and low frequencies will produce bubbles of widely differing sizes. These will not coalesce to the same extent as more uniformly size-distributed bubbles due to Bjerknes forces [28]. Bubble coalescence encourages the formation of degassing bubbles; therefore, the relative absence of coalescence during dual frequency sonication will lead to a higher proportion of cavitating bubbles, which will undergo vigorous and efficient collapse. Also, dual frequencies enhance the formation of daughter bubbles which serve as nucleation sites for more bubbles to be formed, and hence the sonochemical activity is enhanced [29].

Low-frequency cavitation regimes generate cavitation bubbles that collapse vigorously. These bubbles have extended lifetimes, reducing the likelihood of ^●^OH radicals escaping from them and entering bulk solution as there is a higher chance of these radicals undergoing recombination before escaping into the bulk. In contrast, at higher frequencies, the bubbles' lifetimes are significantly shorter, allowing more ^●^OH to escape and enter the bulk solution before undergoing any further reactions within the bubble. The increased escape of ^●^OH to the bulk solution at higher frequency helps to increase the chance of further oxidation reactions occurring [25]. When ^●^OH interact with methylene blue, the formation of intermediate products such as benzene sulfonic acid derivatives and phenols results. Additionally, the combination of ^●^OH radicals to form H_2_O_2_ can effectively boost the efficiency of methylene blue degradation according to the reactions illustrated in equations (4–6) [30], [31].

H_2_O → ^●^OH + ^●^H (4)

2^●^OH → H_2_O_2_ (5)

Methylene blue + H_2_O_2_ → *x*CO_2_ + *y*H_2_O + inorganic ions (6)

Therefore, with the combination of low and high frequencies, the quality of cavitation bubbles with regard to the degradation of methylene blue was increased.

Secondly, the performance of dual frequency operation showed a notable enhancement when the frequency difference was larger. Specifically, there was substantial improvement with dual frequencies of 20 and 80 kHz compared to 20 and 37 kHz, as observed in regime 3 of Table 1. This is due to the improved chemical effects resulting from the combination of two significantly different mean bubble sizes. At the higher frequency of 80 kHz, the bubbles’ lifetimes are considerably shorter compared to those at 37 kHz. Therefore, it is anticipated that a greater number of ^●^OH radicals escape from the bubbles into the bulk solution before substantial recombination occurs within the bubbles. Consequently, the pairing of 20 and 80 kHz frequencies generates high-quality cavitation bubbles, leading to enhanced degradation efficiency, as indicated in Table 1.

### 3.3 Effect of frequency difference on the degradation efficiency

In this section, we present the impact of frequency difference on the degradation efficiency of methylene blue. The objective is to operate both acoustic devices (ultrasonic horn and bath) at their maximum capacity for 20 min to assess their effectiveness in degrading methylene blue (i.e., horn at 20 kHz operating at 80 % power with bath at 37 kHz operating at 100 %; horn at 20 kHz operating at 80 % power with bath at 80 kHz operating at 100 % power). It is important to highlight that the ultrasonic horn was not operated beyond 80 % of its input power due to safety concerns and performance inconsistencies [27]. The degradation performance was evaluated at 5, 10, 15, and 20 min, and the results are tabulated in Table 2.

**Table 2:** The performance of two distinct dual frequencies (20 and 80 kHz; and 20 and 37 kHz).

**Time [minutes]**	**Dual frequencies (20 and 80 kHz)**, **acoustic power density (209.63 ± 6.94 W/L)**		**Dual frequencies (20 and 37 kHz),acoustic power density (272.08 ± 3.92 W/L)**	
	Final MB Concentration [µM]	Percentage Degradation [%]	Final MB Concentration [µM]	Percentage Degradation [%]
5	3.77 ± 0.0036	74.86	3.70 ± 0.0045	75.35
10	2.42 ± 0.0039	83.87	2.84 ± 0.0019	81.04
15	1.93 ± 0.0020	87.12	1.95 ± 0.0088	87.02
20	1.35 ± 0.0007	91.00	1.91 ± 0.0022	87.29

**Fig. 6:**
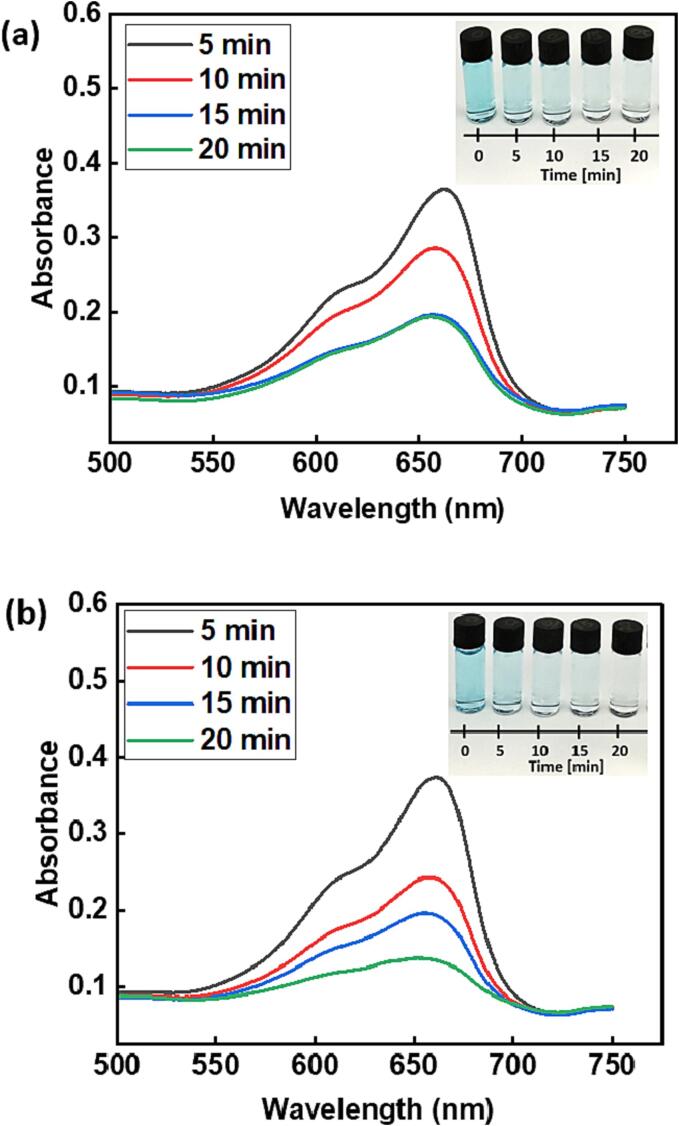
The color degradation of 15 µM (4.8 mg/L) methylene blue solution under dual frequency irradiation: a) 20 and 37 kHz, b) 20 and 80 kHz.

The results from Table 2 show that the degradation efficiency is generally higher with dual frequencies 20 and 80 kHz as compared with that achieved using 20 and 37 kHz, despite the acoustic power density of the latter condition (272.08 **±** 3.92 W/L) being higher than that from the former (209.63 **±** 6.94 W/L). The degradation of the methylene blue’s color alongside the UV–vis spectra taken at intervals of 5 min is shown in Fig. 6.

**Fig. 7:**
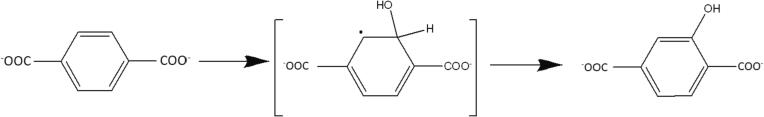
Outline pathway for the formation of 2-hydroxyterephthalate in the presence of ^●^OH radicals.

To further illustrate the effect of generated ^●^OH and H_2_O_2_ on the sonochemical degradation of methylene blue during sonication, the concentration of both were quantified at specific ultrasonic treatment intervals. In this study, the production of ^●^OH was quantified using dosimetry methods described in references [32] and [33]. Terephthalic acid generates terephthalate anions in an alkaline aqueous solution. Terephthalate anions subsequently undergo reaction with ^●^OH radicals to yield intensely fluorescent 2-hydroxyterephthalate anions (HTA) as illustrated in Fig. 7. The concentration of these 2-hydroxyterephthalate anions was then determined through fluorescence measurements. On the other hand, H_2_O_2_ was quantified using iodometry techniques as described in references [34] and [35]. In this method, iodide ions (I^−^) are oxidized by H_2_O_2_ in the presence of a molybdate catalyst to form iodine (I_2_). In turn, iodine reacts with excess iodide to form triiodide (I_3_^−^), which has a strong ultraviolet absorbance peak at 350 nm (ε ≈ 26,000 L mol^−1^ cm^−1^) [36]. Hence, the triiodide concentration was used to ascertain the amount of H_2_O_2_ generated in a given treated sample. Since the presence of ^●^OH and H_2_O_2_ led to the facile degradation of methylene blue, the concentrations of both ^●^OH and H_2_O_2_ were determined over time in the treated samples at both 37/20 kHz and 80/20 kHz. The ^●^OH radical and H_2_O_2_ concentrations increase with time under both experimental regimes. During a 20-minute ultrasonic irradiation at respective dual frequencies of 37/20 kHz and 80/20 kHz, concentrations of ^●^OH / H_2_O_2_ of 135.12 nM / 56.73 µM and 150.29 nM / 61.89 µM were produced, as indicated in Table 3 (see also Fig S5 and Fig S6 in the Supporting Information for further information and a detailed description of the two methods).

**Table 3:** Showing the concentration of 2-hydroxyterephthalate (HTA), ^●^OH and H_2_O_2_ after sonication times of 5, 10, and 20 min.

**Time [minutes]**	**Dual frequencies (20 and 80 kHz),** a**coustic power density (209.63 ± 6.94 W/L)**			**Dual frequencies (20 and 37 kHz),** a**coustic power density (272.08 ± 3.92 W/L)**		
	**Concentration**			**Concentration**		
	HTA [µM]	^●^OH [nM]	H_2_O_2_ [µM]	HTA [µM]	^●^OH [nM]	H_2_O_2_ [µM]
5	1.50	37.59	17.22	1.84	46.02	23.37
10	3.14	78.51	32.06	2.97	74.15	35.80
20	6.01	150.29	61.89	5.40	135.12	56.73

**Fig. 8:**
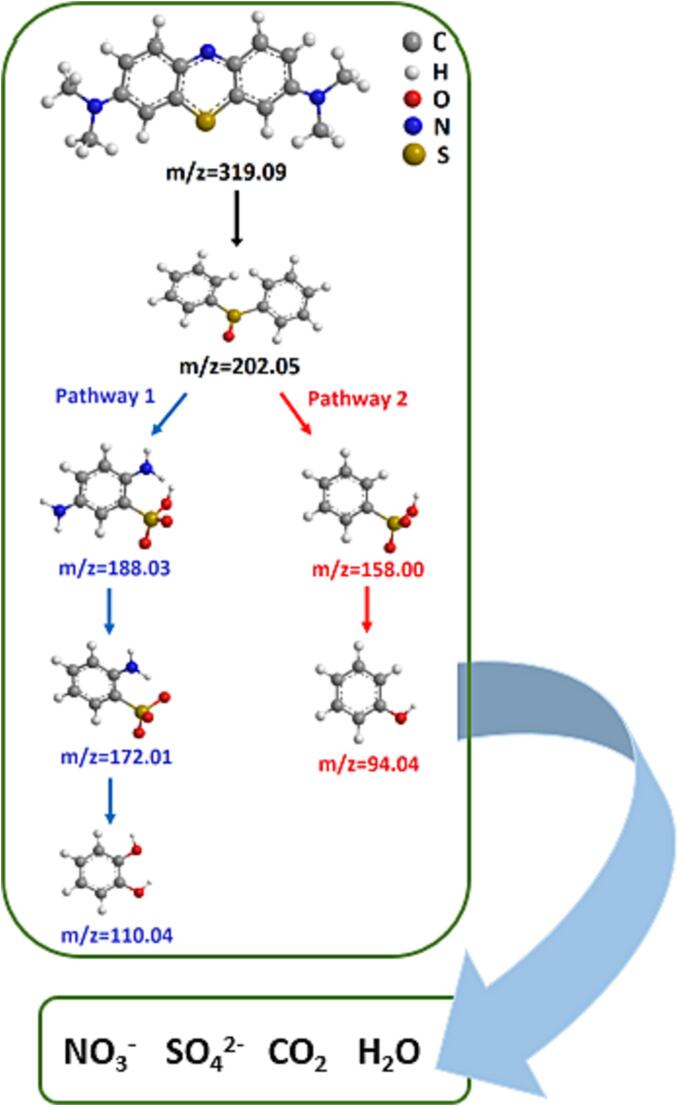
Possible degradation pathway of methylene blue on the basis of mass spectrometry studies.

Fig. 6a and 6b illustrate the color transformation as the sonication period progresses from 0 to 20 min under both dual frequency conditions. Additionally, it is noticeable that the blue color almost disappears after 15 min in both cases. Furthermore, analysis of samples by mass spectrometry showed that a very low-intensity peak corresponding to methylene blue could be detected after 15 min of sonication using either dual frequency combination, see Fig. S4. of the Supplementary Information. Based on the results from mass spectrometry (see also Fig S4 (a-f) in the Supplementary Information) at different sonication times (0, 5 and 15 min), a degradation pathway was deduced as shown in Fig. 8, which is in agreement with that reported previously [30], [37]. In this degradation pathway, the principal active species responsible for the degradation of methylene blue are proposed to be ^●^OH radicals and H_2_O_2_. Following the initiation of the degradation process through attack on the N-S heterocyclic ring by free radicals, two distinct pathways are discernible, yielding intermediate benzenesulfonic acid derivatives. In the first pathway, 2-aminobenzenesulfonate is produced, followed by the subsequent removal of the amino group, resulting in the formation of 1,2-benzenediol. In the second pathway, benzenesulfonic acid undergoes further reaction to produce phenol. In both pathways, the intermediate benzene ring undergoes ^●^OH attack and oxidation due to H_2_O_2_, ultimately leading to the mineralization of methylene blue and its degradation products into simpler inorganic molecules.

**Fig. 9:**
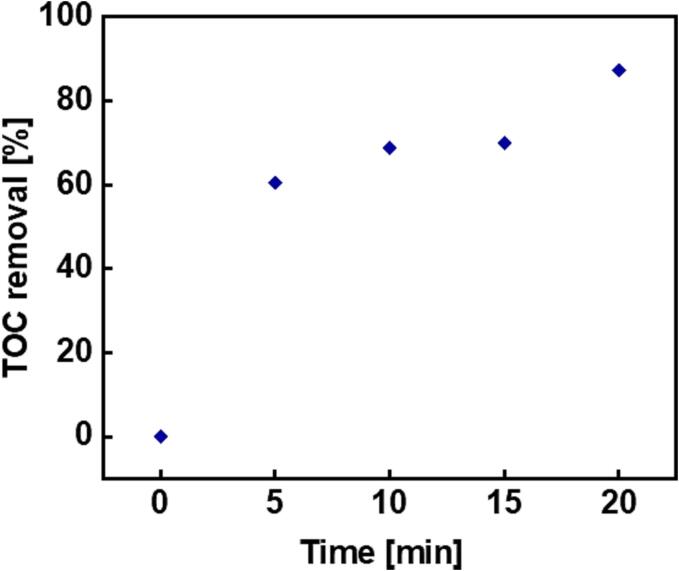
The total organic carbon removal efficiency as a function of sonication time using dual frequencies of 20 and 80 kHz.

The total organic carbon (TOC) content is another parameter commonly used to evaluate the efficiency of organic pollutant degradation in wastewater treatment [38]. Therefore, further analysis of treated solutions for their total organic carbon using an organic carbon analyser (Thermalox Elemental analyser, with model number TOC-2020) revealed the degree to which organic carbon was removed from the solution following specific ultrasonic treatments employing dual frequencies of 20 and 80 kHz (horn and bath respectively). The TOC removal efficiency was calculated using equation (7) and is shown in Fig. 9.

(7) $$TOC\;\;removal\;\;efficiency\;=\frac{{\mathrm{TOC}}_{0}-TOC}{{\mathrm{TOC}}_{0}}\;\times 100\%$$

where *TOC_0_* and *TOC* are the initial and final concentration of total organic carbon present before and after ultrasonic treatment [38]. Fig. 9 shows a total organic carbon removal efficiency of 87.42 % after just 20 min of dual frequency ultrasound treatment at an acoustic power density of 209.63 ± 6.94 W/L.

### 3.4 Kinetics and rate constants of dual-frequency removal of methylene blue

**Fig. 10:**
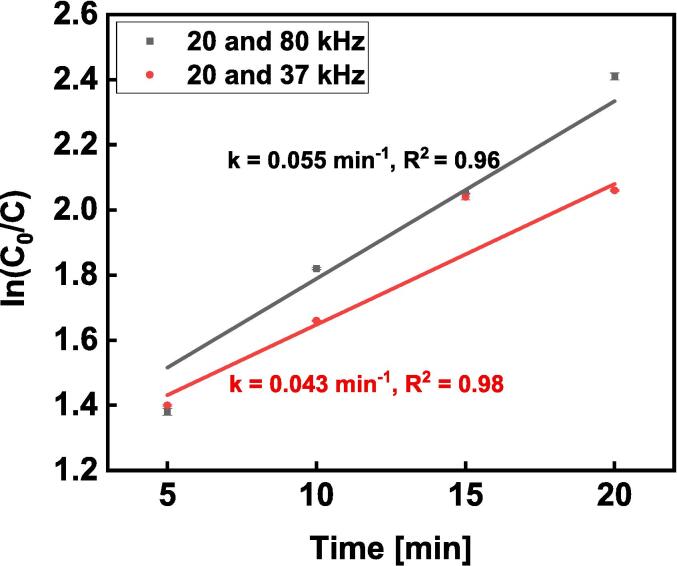
The plot of ln (*C_0_*/*C*) against time (min) for degradation of methylene blue under dual frequency conditions (20/37 and 20/80 kHz).

The kinetics of dual-frequency degradation of methylene blue were explored by employing the Langmuir–Hinshelwood kinetic model [31], as illustrated by the following equation:

(8) $$ln\left(\frac{C_{o}}{C}\right)=kt+B$$

where *B* is a constant, *k* is the degradation rate constant (min^−1^), *t* is the reaction time, and *C_0_* and *C* are the respective initial concentration and the final concentration after time *t* of ultrasonic treatment. The mean and standard deviation of ln (*C_0_*/*C*) from three samples under each experimental regime were plotted against the treatment time *t* (min), and the curves were fitted using equation (8) as shown in Fig. 10. The rate constant, *k* was estimated from the slopes of the plots. The rate constants were found to be 0.055 min^−1^ and 0.043 min^−1^ for the respective 20/80 and 20/37 kHz dual-frequency treatments. The corresponding regression coefficient (the square of the relative correlative coefficients (R^2^)) of the experimental runs indicate that the degradation of methylene blue using dual-frequency ultrasound followed first order kinetics.

The comparison of the performance of this method with those reported for other techniques in the literature is shown in Table 4. The data shows that this dual frequency approach holds promise for methylene blue degradation in terms of the power density required to achieve a given level of degradation.

**Table 4:** Comparison of the performance between the dual frequency method reported herein and those of alternative techniques reported in the literature with added catalysts/additives for the degradation of methylene blue.

Method	Power density [W/L]	Treatment time [min]	Degradation efficiency [%]	Reference
• Ultrasound plus CCl_4_ • Ultrasound alone	5000 _	35 30	98.6 36.75	Li et al. [12]
• Ultrasound with H_2_O_2_ and TiO_2_ • Ultrasound alone	–	60	85 25	Shimizu et al. [13]
• Ultrasound with TiO_2_ • Sonophotodegradation	_ _	180	30 45	Yuan et al.[14]
Ultrasound with CaMgO_2_ under sunlight	–	60	95	Karuppusamy et al.[15]
Ultrasound with rGO/TiO_2_ under sunlight	–	30	91.3	Deshmukh et al. [16]
Ultrasound with Ag-ZnO nanocomposite	_	120	96.2	Satdeve et al, [17]
Ultrasound (490 kHz) with TiO_2_	80	30	–	Honma et al, [39]
Ultrasound with CeO_2_	800	120	90	Gadge et al, [31]
Ultrasound with β-NiMoO_4_ under sunlight	2000	150	98.2	Dhanasekar et al, [23]
Ultrasound only (20 and 37 kHz)	272.08 ± 3.92	20	87.29	This work
Ultrasound only (20 and 80 kHz)	209.63 ± 6.94	20	91	This work

## 4 Conclusions

Enhanced sonochemical degradation efficiency of methylene blue can be achieved by improving the quality of cavitation-mediated bubbles without the need for additives or catalysts. This paper presents an efficient method for enhancing the quality of acoustically generated cavitation bubbles using dual frequencies (20/37 kHz; and 20/80 kHz). The effect of the difference in the frequency used was investigated, and it was observed that the larger the difference in the dual frequencies used, the more significant the cavitation-mediated effect on methylene blue degradation. After ultrasonic treatment for 20 min using dual frequencies of 20/80 kHz, a degradation efficiency of 91 % for methylene blue was achieved. Therefore, this method can be considered an effective, environmentally friendly, additive/catalyst-free technique for the degradation of methylene blue.

## CRediT authorship contribution statement

**Lukman A. Yusuf:** Conceptualization, Data curation, Investigation, Methodology, Writing – original draft. **Zeliha Ertekin:** Investigation, Methodology, Writing – review & editing. **Shaun Fletcher:** Investigation, Methodology. **Mark D. Symes:** Conceptualization, Supervision, Writing – review & editing.

## Declaration of competing interest

The authors declare the following financial interests/personal relationships which may be considered as potential competing interests: Mark Symes reports financial support was provided by EPSRC.

## Data Availability

The data underpinning this study have been deposited in the University of Glasgow’s Enlighten database under accession code https://doi.org/10.5525/gla.researchdata.1590.
