# Supplementary material for: Enhanced ultrasonic degradation of methylene blue using a catalyst-free dual-frequency treatment
Source: Ultrason Sonochem. 2024 Feb 3;103:106792. doi: 10.1016/j.ultsonch.2024.106792 (PMC10878993; doi:10.1016/j.ultsonch.2024.106792)
Supplement: Supplementary data 1 [file mmc1.docx]

**Enhanced ultrasonic degradation of methylene blue using a catalyst-free dual-frequency treatment**

*Lukman A. Yusuf,^a^*  *Zeliha Ertekin,^a.b^ Shaun Fletcher,^a.^ and Mark D. Symes^a*^*

*^a^ WestCHEM, School of Chemistry, University of Glasgow, University Avenue, Glasgow, G12 8QQ, United Kingdom.*

*^b^ Hacettepe University, Faculty of Science, Department of Chemistry, Beytepe, 06800 Ankara, Turkey.*

** Email:* [mark.symes@glasgow.ac.uk](mailto:mark.symes@glasgow.ac.uk) *(M. D. Symes)*

**Fig S1.** Calibration curve from different standard solutions of methylene blue. The standard solutions (8 – 41 µM, equivalent to 2.56 – 13.1 mg/L) were prepared from a stock solution of 0.1 mM. These solutions were analysed by UV-vis (Agilent Cary 60 UV-vis spectrometer), and the absorbance values at a wavelength of 664 nm were recorded.


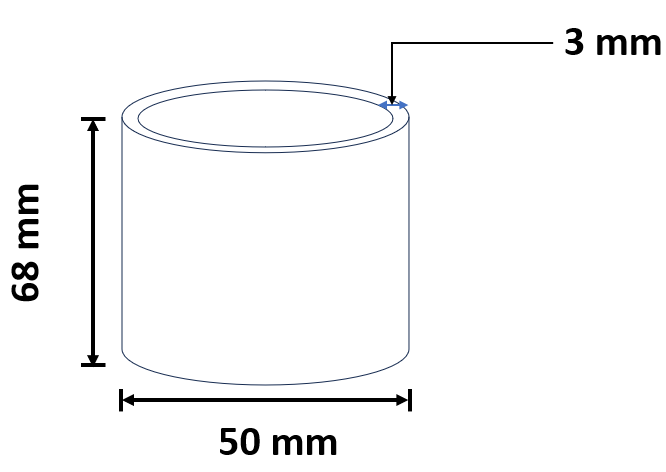


**Fig. S2.** Schematic diagram of the sonoreactor beaker used for the experiments. This reactor was built in-house from glass and was used throughout all the experiments.


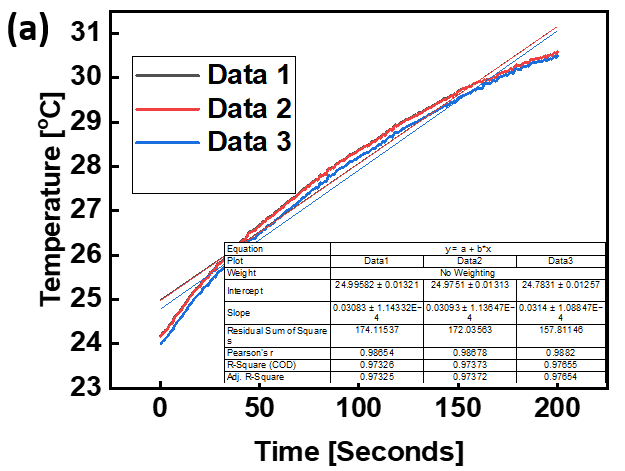


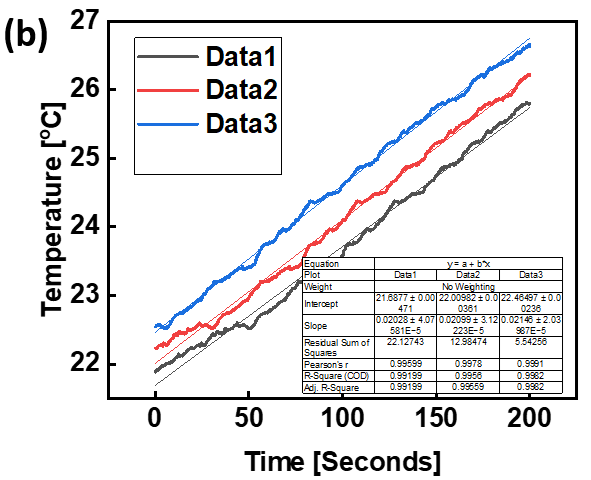


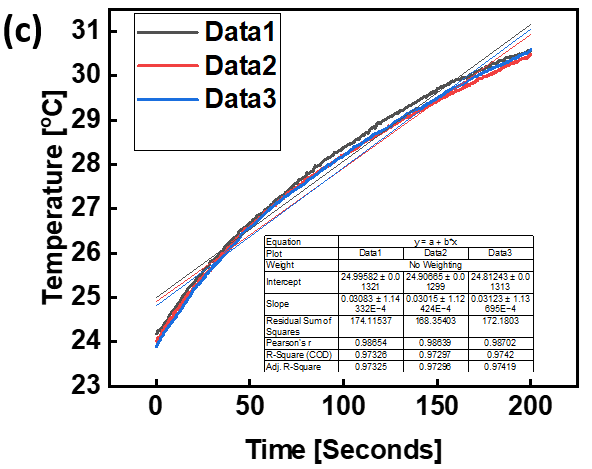


**Fig. S3.** Calorimetric data obtained from the temperature rise per second when (a) the ultrasonic bath was operated at frequency and input power of 37 kHz and 100% respectively; (b) the ultrasonic bath was operated at frequency and input power of 80 kHz and 100% respectively; and (c) the simultaneous combined operation of the ultrasonic horn (at 37 kHz and 60% input power) and the ultrasonic horn at 40% input power.


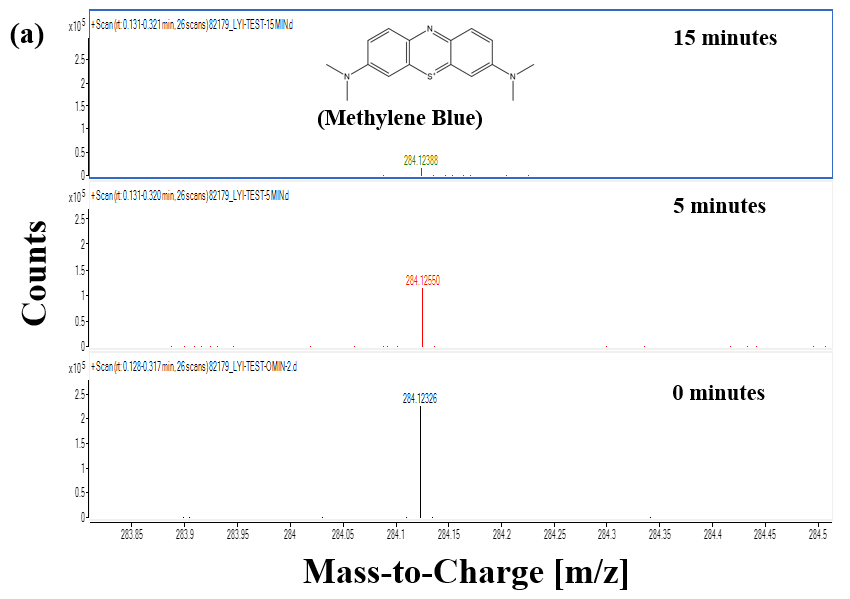


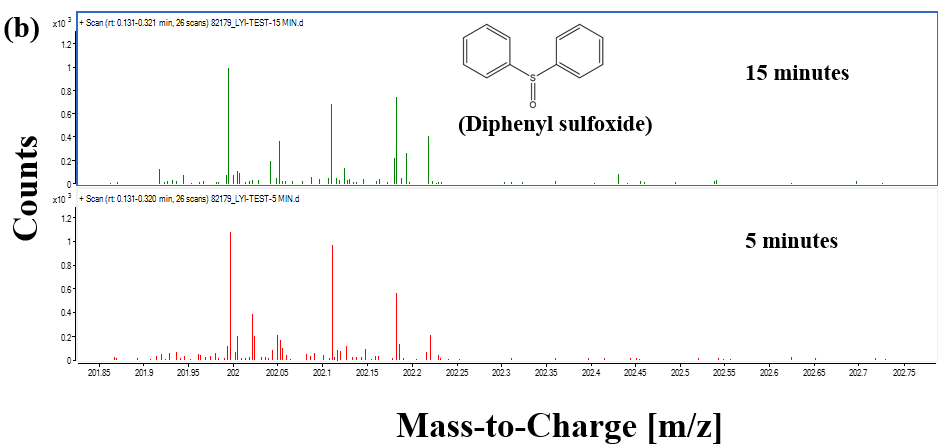


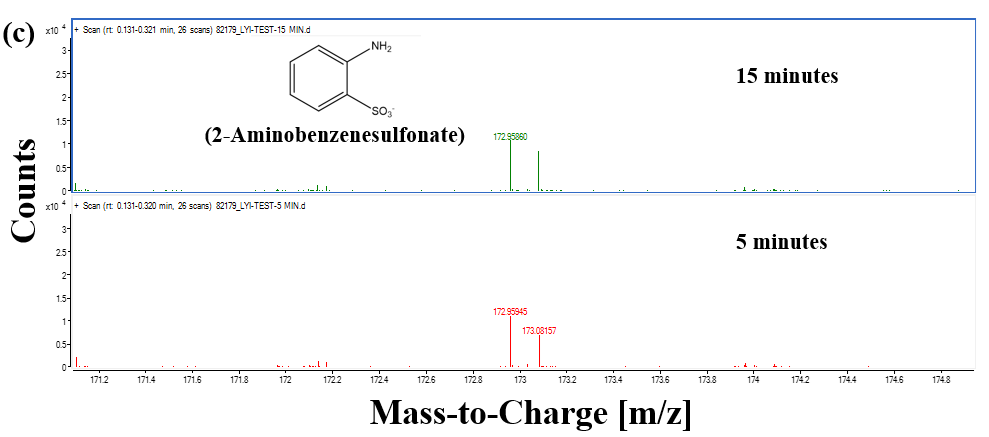


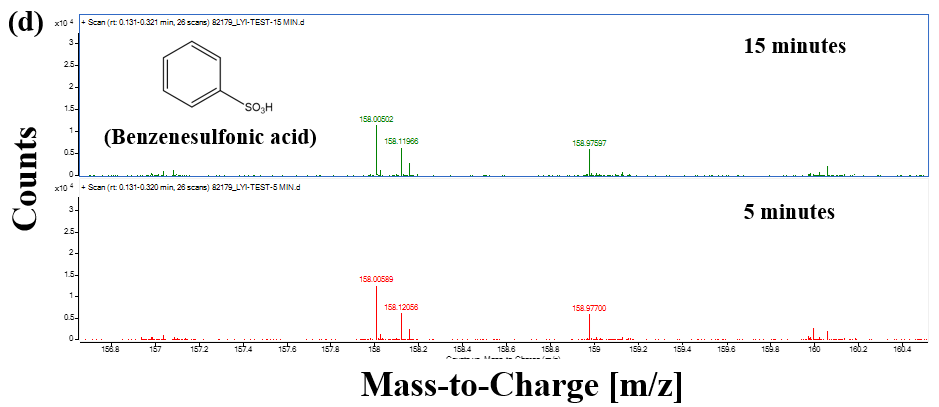


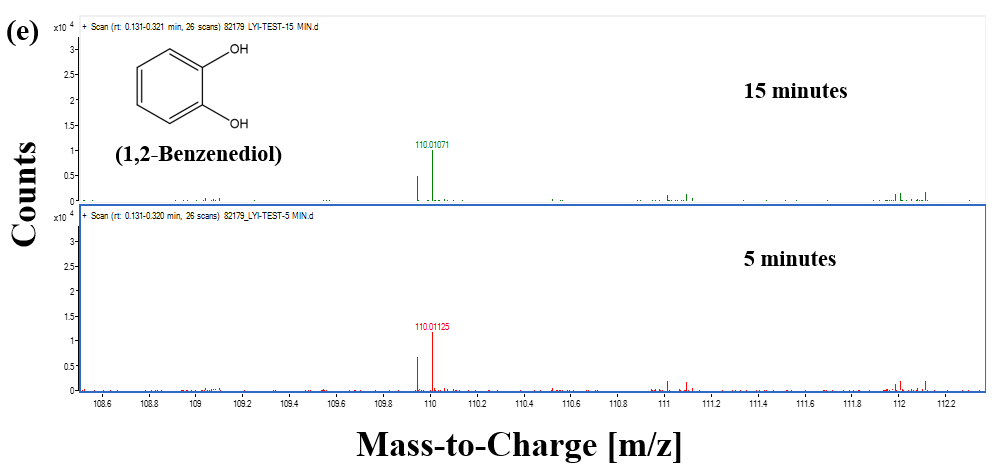


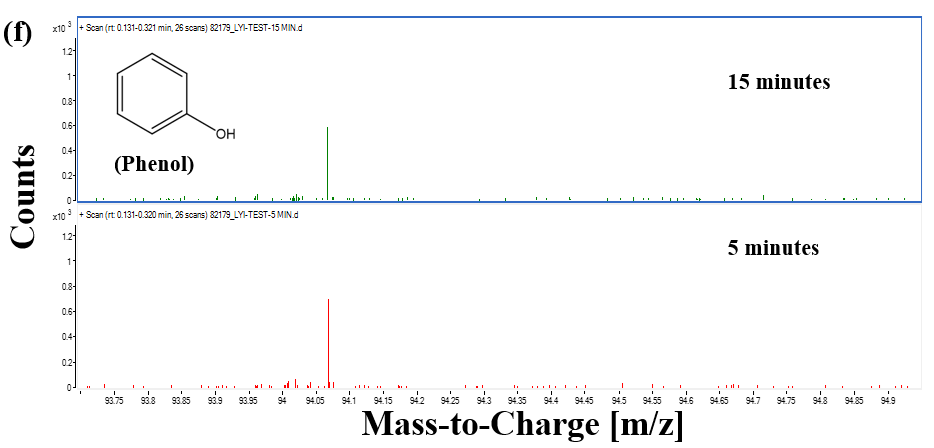


**Fig. S4.** Mass spectrometry data for methylene blue: (a) shows the extent of degradation of methylene blue after 5 and 15 minutes, (b-f) show the traces of the by-products as the sonication progresses.


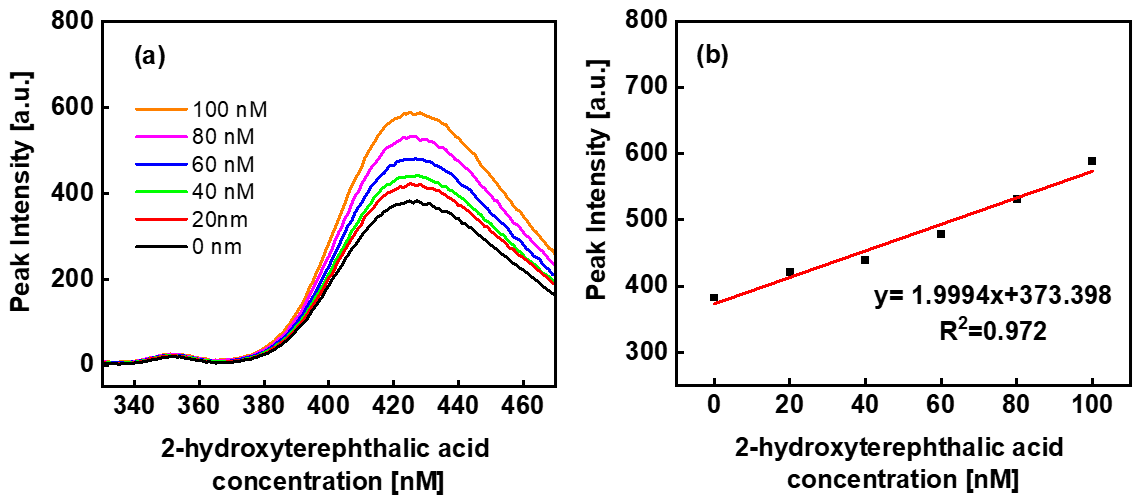


**Fig. S5.** Fluorescence spectra (a) and the resulting calibration curve (b) for 2-hydroxyterephthalic acid in alkaline aqueous solution with NaOH (2.5 × 10^−3^ mol) and phosphate buffer including KH_2_PO_4_ (2.2 × 10^−3^ mol) / Na_2_HPO_4_ (3.5 × 10^−3^ mol). The fluorescence of each solution was measured using a Perkin-Elmer Cary Eclipse Fluorescence spectrophotometer, employing an excitation wavelength of 315 nm and an analyzing wavelength of 425 nm; the emission wavelength ranged from 330 to 470 nm with a 10 nm excitation slit and a 10 nm emission slit, at a data acquisition rate of 600 nm per minute.


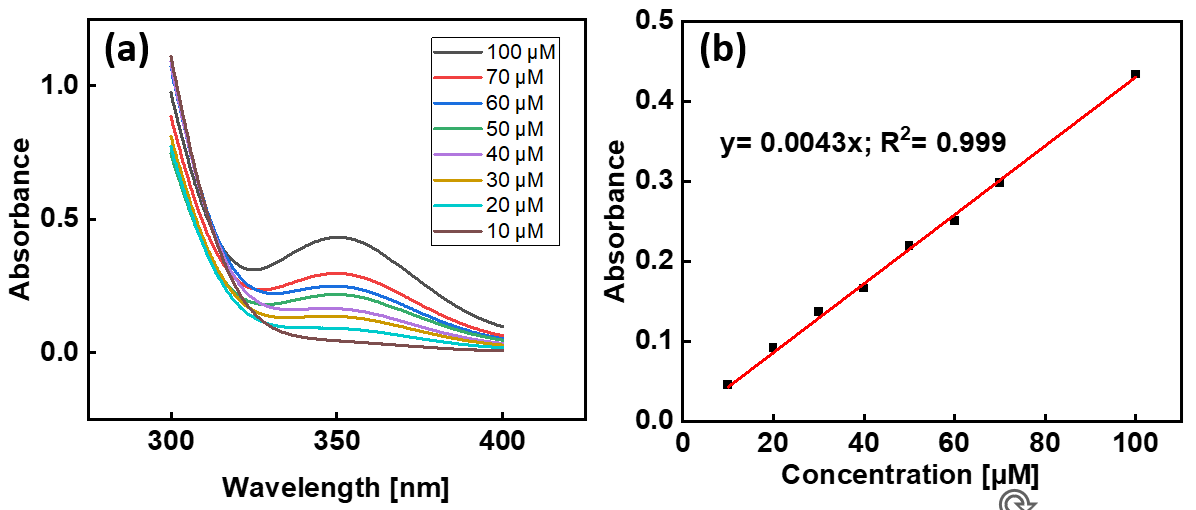


**Fig. S6.** (a) UV spectra from a standard solution and (b) the resulting plot of absorbance at 350 nm against standard solution concentration, which serves as the calibration curve for iodometric tests on standard solutions of hydrogen peroxide (10 – 100 μM, prepared from a 30% w/w stock solution of hydrogen peroxide in water). 600 μL of each standard solution was combined with 3 mL of potassium iodide solution (0.1 M) and 60 μL of ammonium molybdate solution (0.01 M) in a quartz cuvette. After five minutes the mixture was analysed by UV-vis (Agilent Cary 60 UV-vis spectrometer), and the absorbance at 350 nm was recorded.
